# Deletion Hotspots in *AMACR* Promoter CpG Island Are *cis*-Regulatory Elements Controlling the Gene Expression in the Colon

**DOI:** 10.1371/journal.pgen.1000334

**Published:** 2009-01-16

**Authors:** Xiang Zhang, Irwin Leav, Monica P. Revelo, Ranjan Deka, Mario Medvedovic, Zhong Jiang, Shuk-Mei Ho

**Affiliations:** 1 Department of Environmental Health, University of Cincinnati College of Medicine, Cincinnati, Ohio, United States of America; 2 Center for Environmental Genetics, University of Cincinnati College of Medicine, Cincinnati, Ohio, United States of America; 3 Department of Surgery, University of Massachusetts Medical School, Worcester, Massachusetts, United States of America; 4 Department of Cancer Biology, University of Massachusetts Medical School, Worcester, Massachusetts, United States of America; 5 Department of Pathology and Laboratory Medicine, University of Massachusetts Medical School, Worcester, Massachusetts, United States of America; 6 Department of Pathology and Laboratory Medicine, University of Cincinnati College of Medicine, Cincinnati, Ohio, United States of America; 7 Center for Genome Information, University of Cincinnati College of Medicine, Cincinnati, Ohio, United States of America; 8 Cancer Center, University of Cincinnati College of Medicine, Cincinnati, Ohio, United States of America; University of Illinois at Urbana-Champaign, United States of America

## Abstract

Alpha-methylacyl-coenzyme A racemase (AMACR) regulates peroxisomal β-oxidation of phytol-derived, branched-chain fatty acids from red meat and dairy products — suspected risk factors for colon carcinoma (CCa). AMACR was first found overexpressed in prostate cancer but not in benign glands and is now an established diagnostic marker for prostate cancer. Aberrant expression of AMACR was recently reported in Cca; however, little is known about how this gene is abnormally activated in cancer. By using a panel of immunostained-laser-capture-microdissected clinical samples comprising the entire colon adenoma–carcinoma sequence, we show that deregulation of *AMACR* during colon carcinogenesis involves two nonrandom events, resulting in the mutually exclusive existence of double-deletion at CG3 and CG10 and deletion of CG12-16 in a newly identified CpG island within the core promoter of *AMACR*. The double-deletion at CG3 and CG10 was found to be a somatic lesion. It existed in histologically normal colonic glands and tubular adenomas with low AMACR expression and was absent in villous adenomas and all CCas expressing variable levels of AMACR. In contrast, deletion of CG12-16 was shown to be a constitutional allele with a frequency of 43% in a general population. Its prevalence reached 89% in moderately differentiated CCas strongly expressing AMACR but only existed at 14% in poorly differentiated CCas expressing little or no AMACR. The DNA sequences housing these deletions were found to be putative *cis*-regulatory elements for Sp1 at CG3 and CG10, and ZNF202 at CG12-16. Chromatin immunoprecipitation, siRNA knockdown, gel shift assay, ectopic expression, and promoter analyses supported the regulation by Sp1 and ZNF202 of *AMACR* gene expression in an opposite manner. Our findings identified key *in vivo* events and novel transcription factors responsible for *AMACR* regulation in CCas and suggested these *AMACR* deletions may have diagnostic/prognostic value for colon carcinogenesis.

## Author Summary

Men consuming high amounts of red meat and dairy products are at a higher risk of developing colon and prostate cancer. Alpha-methylacyl-coenzyme A racemase (AMACR) is an enzyme that helps to break down fat from these foods to produce energy. An increase in the utilization of energy from fat is a hallmark of many cancers including colon and prostate cancers. Indeed, the *AMACR* gene was first found to be abnormally active in prostate cancers, and its abnormal expression has become a diagnostic marker for the cancer. However, little is known about how *AMACR* becomes activated in cancer cells. Here, we show that *AMACR* is also highly expressed in certain stages of colon cancer, though not all stages. A close examination of the *AMACR* gene in a panel of normal and progressively malignant colon tissues reveals that deletions of specific sequences in the *AMACR* gene may trigger its abnormal expression during the evolution of colon cancer. We also identify unique proteins known as “transcription factors” that normally bind to these deleted sequences to maintain normal expression of the gene. Finally, we report a new deletion variant of the *AMACR* gene in the general population that may influence the course of colon carcinogenesis.

## Introduction

Alpha-methylacyl-CoA racemase (AMACR) is a peroxisomal and mitochondrial enzyme that is indispensable in the catabolism of phytol-derived, 2-methyl-branched-chain fatty acids and the synthesis of bile acids [1]. In hepatocytes, AMACR catalyzes the conversion of pristanoyl-CoA and C27-bile acyl-CoAs from R- to S-stereoisomers, which are the only stereoisomers that can undergo β-oxidation. Bile acid intermediates undergo one round of β-oxidation in the peroxisomes and are secreted. In contrast, branched-chain fatty acid derivatives are transported to mitochondria, where they are further degraded to generate biological energy. Since most malignancies increase fatty acid utilization as an energy source to fuel growth [2], it has been suggested that increased β-oxidation of branched-chain fatty acids provides transformed cells with a unique metabolic advantage [3]. This idea is supported by recent findings that knockdown of *AMACR* transcripts or inhibition of the racemase activity effectively blocked growth of prostate cancer (PCa) cells [4],[5]. In humans, the major sources of phytol-derived, 2-methyl-branched fatty acids are dietary ruminant fats, meat, and dairy products. Increased consumption of these foods are known risk factors for prostate and colon carcinoma (CCa) [6],[7].

Aberrant expression of AMACR was first reported in PCa and high-grade prostatic intraepithelial neoplasia but not in benign hyperplastic lesions or normal epithelia [8],[9]. These findings quickly led to the establishment of AMACR as a reliable diagnostic marker for PCa [10]–[13]. More recently, overexpression of AMACR also was reported in CCa [14]–[18], with a prevalence between 45% and 75% [19]–[21]. However, the relationship between levels of AMACR expression and the sequence of adenoma-carcinoma progression in the colon [22] has not been fully characterized. Except for a report that identified a non-canonical CCAAT enhancer element in the *AMACR* promoter [5] and a lack of regulation of this gene by androgen [16],[23], no information is available regarding how the *AMACR* gene is regulated. Furthermore, although recent studies have identified a few *AMACR* gene variants to be associated with PCa [24],[25] or CCa [26] risks, a sequence polymorphism in the promoter region of *AMACR* has not been reported.

Given the potential significance of AMACR in CCa, our objectives in this study were to determine the mechanisms of *AMACR* gene regulation *in vivo* during neoplastic transformation of the colon epithelium. Through the use of a comprehensive panel of immunostained-laser-capture-microdissected (iLCM) clinical samples comprising the entire colon adenoma-carcinoma sequence, we now report that the deregulation of *AMACR* during colon carcinogenesis involves non-random events, resulting in a double-deletion at CG3 and CG10, and alterations in the frequencies of deletion of CG12-16 in a newly identified CpG island (CGI) located within the core promoter of *AMACR*. We also identified deletion of CG12-16 as a putative regulatory polymorphism and the double-deletion at CG3 and 10 as a somatic lesion. The DNA sequences housing these deletions were indicated to be a *cis*-regulatory element for Sp1 and a putative ZNF202-binding site, respectively, and to exert opposite effects on *AMACR* transcription.

## Results

### Overexpression of AMACR in Villous Adenomas and in Well- and Moderately Differentiated CCas but not in Poorly Differentiated CCas

**Figure 1:**
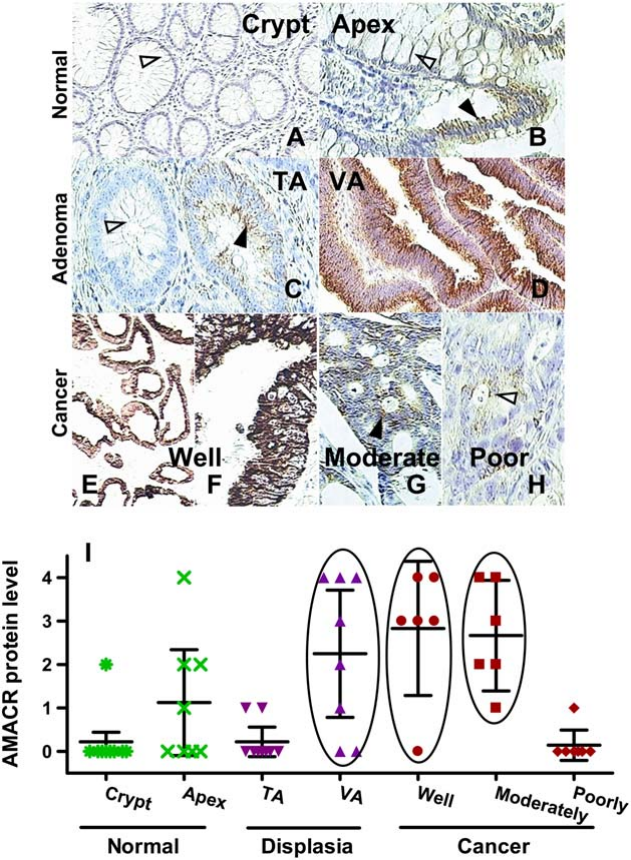
Detection of AMACR expression level by immunostaining. A–H: the typical AMACR immunostaining found in normal and neoplastic colon sections from our case materials. I: AMACR expression scores of these foci, representing the above groups, depicted in a scatter plot. One-way analysis of variance, followed by Tukey's HSD *post hoc* test, indicated the significant difference among different groups (*p*<0.0001). The normal crypt group served as a reference. Foci with normal cryptal glands had very low AMACR expression (score: 0.22±0.22), with 8 of 9 foci scored negative. Foci with normal apical surface epithelium had mildly elevated expression (1.1±0.55) that was not statistically different from that of the former group (*p* = 0.67). Expression at foci harboring TA glands with a mild degree of dysplasia (0.22±0.15, *p* = 1.00) was not statistically different from that in normal cryptal glands. However, VAs had elevated expression (2.3±0.62, *p* = 0.007) comparable to that of well- (2.8±0.60, *p* = 0.001) and moderately differentiated carcinomas (2.7±0.49, *p* = 0.002); the three groups (open ellipses) have higher AMACR expression scores than normal and TAs. In marked contrast, AMACR expression scores in poorly differentiated cancers were low (0.14±0.14, *p* = 1.00), with 6 of 7 foci devoid of AMACR immunostaining.

We first provided a detailed description of the relationship between AMACR expression levels and the sequence of adenoma-carcinoma progression in the colon. The levels of AMACR in 55 foci representing seven normal, premalignant and malignant histological entities in 35 colon specimens were semiquantified in immunostained slides (Figure 1A to 1H). These foci were subsequently microdissected for *AMACR* promoter studies. In general, AMACR immunostaining was negative to weak in normal cryptal (Figure 1A) and apical (Figure 1B) epithelia, as well as in tubular adenomas (TAs) with mild dysplasia (Figure 1C). In contrast, villous adenomas (VAs) (Figure 1D), well- (Figure 1E and 1F) and moderately (Figure 1G) differentiated adenocarcinomas expressed high levels of AMACR. AMACR immunostaining was almost absent to negligible in poorly differentiated carcinomas (Figure 1H). Compared with the expression in normal crypt, levels of AMACR expression, represented as a score of 0 to 4, were significantly increased in VAs and in well- and moderately differentiated carcinoma but not in normal apex, TAs, and poorly differentiated carcinoma (Figure 1I).

### Organization of the AMACR Proximal Promoter Region

**Figure 2:**
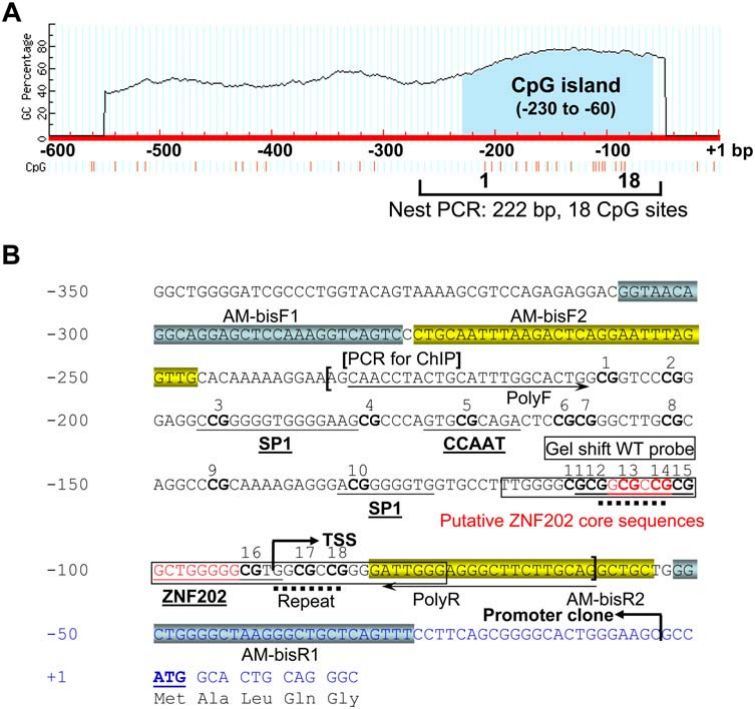
The organization of *AMACR* gene 5′-flanking region. A: The location of the CpG island upstream translation start site (designated as +1). Individual CG sites are indicated as red vertical lines and numbered from 1 to 18. The 222-bp nested PCR-amplified CGI is illustrated. B: Partial exon 1 and the promoter sequence encompassing the CGI. The first exon is indicated by a bent arrow (TSS). Predicated transcription factor binding sites of Sp1 and ZNF202, together with the CCAAT enhancer binding site, are underlined. The locations of two pairs of primers for bisulfite sequencing PCR are blocked with different colors. The ChIP assay-amplified region is marked with brackets. The DNA upstream -4 (bent arrow, promoter clone) was cloned for promoter analysis. Two direct repeats of up to 7 nt (5′-GGCGCCG-3′) that may related to the deletion caused by slipped-strand mispairing are marked by dotted lines. Primers PolyF/PolyR for polymorphism study are marked with the arrows. The wild type (WT) probe for gel shift assay targeting on putative ZNF202 binding site is boxed. Two short putative ZNF202 core sequences identified by MatInspector were highlighted in red.

Because virtually no information is available on how *AMACR* is regulated *in vivo*, we initially were interested in determining if changes in DNA methylation status of the *AMACR* 5′ flanking promoter region play a role in gene regulation. *In silico* analysis revealed that *AMACR* transcripts share the same first exon with an 88-bp 5′ untranslated region (5′ UTR), suggesting that the gene is controlled by one promoter. Two CGIs were identified flanking the transcription start site (Figure 2A). The first is a novel CGI located upstream of the ATG site (−230 to −60; the position of the translation start site was set as +1) with 18 CG dinucleotides, whereas the second CGI downstream of the ATG site (48 to 357, not shown in Figure 2A) has been reported and shown to not be involved in gene regulation in PCa cells [5]. In concordance, our pilot studies indicated that the downstream CGI exhibited no differences in methylation/deletion/mutation status among the histological entities of the colon (data not shown). Hence, subsequent studies were focused on analyses of the previously not reported proximal CGI in the *AMACR* promoter region (the *AMACR* promoter CGI). Our bisulfite sequencing data did not support the involvement of DNA methylation of this newly identified CGI in *AMACR* gene regulation *in vivo*, since the promoter is largely unmethylated in all 55 iLCM samples (next section). However, *in silico* analyses identified two putative Sp1 binding sites at CG3 and CG10 and a non-canonical ZNF202 [27] *cis*-element at CG12-16 of this CGI (Figure 2B). Variable frequencies of deletions were found at these sites and later shown to be involved in gene regulation (next section). A previously reported non-canonical CCAAT enhancer element [5] was aligned to CG5. Two direct repeat sequences, 7 bp in length, were noted to flank the transcription start site. We later proposed that these two repeated sequences are involved in the generation of the CG12-16 deletion (dotted lines; see Discussion below).

### Identification of Deletion Hotspots in the Novel *AMACR* Promoter CGI in Colon Tissues

**Figure 3:**
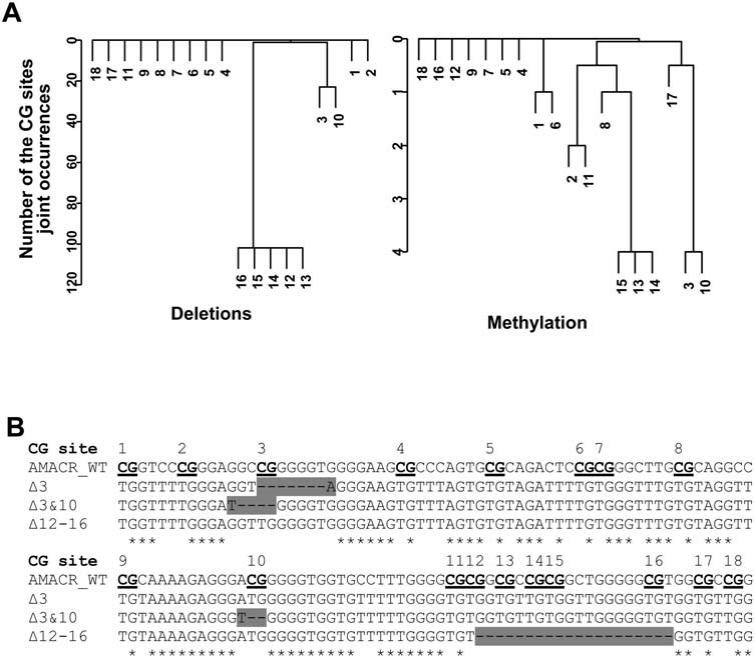
Bisulfite sequencing analysis of iLCM. The results were summarized from the status of 4302 CG sites from a total of 239 alleles in the entire set of 55 microdissected samples. A: Cluster analyses of the deletion and methylation to establish clusters of CG sites based on the entire sequencing data set. The average linkage was employed for hierarchical clustering (sites 1–18). The absolute number of co-occurrences of different CG deletions was used as the similarity measure. The higher the number of clones with two specific CG deletions, the closer they are in the dendrogram. The hotspots were restricted to CG3, 10, and 12-16. B: Typical bisulfite sequencing results of *AMACR* CGI with deletions highlighted in grey. The sequences of *AMACR* promoter variants have been deposited in Genbank and are described in Materials and Methods.

A 222-bp region encompassing all 18 CG sites in the newly identified *AMACR* promoter CGI (Figure 2) was analyzed for methylation, deletion, and mutation changes using DNA obtained from LCM samples and CCa cell lines. Bisulfite sequencing analyses of 239 alleles from 55 foci and regular DNA sequencing of 37 alleles from 9 foci as the control (also see next section) showed that most of the CG sites were unmethylated (Table 1). However, variable frequencies of deletions, methylation, and mutations were found to occur almost invariably at CG3, CG10, and CG12-16, with deletions as the predominant lesion among all aberrations. The sequences of these deletion and mutation variants were deposited to Genbank with the accession number from EF636492 to EF636496. Cluster analyses demonstrated that deletion of CG12-16 was the most common co-occurrence, followed by deletion at CG3 and CG10 (double-deletion at CG3 and CG10) (Figure 3A). Cluster analyses data for methylation (Figure 3A), mutations (Figure S1), and all aberrations (Figure S1) were also obtained. The number of deleted nucleotides (nts) was 2 to 8 nts at CG3 and 2 nts at CG10 (Figure 3B). Deletion at CG12-16 was found to be precisely 20 nts. Among the four CCa cell lines examined, CG12-16 deletions were found in SW480 and SW620; no double-deletion of CG3 and 10 was detected in any of these cell lines. Thus, while methylation of this novel CGI does not appear to play a role in gene regulation, deletions of specific sequences or deletion hotspots within this sequence were identified and might play critical roles in the regulation of gene expression and/or the adenoma-carcinoma progression.

**Table 1:** Bisulfite sequencing analysis of iLCM samples.

CG site	01	02	03	04	05	06	07	08	09	10	11	12	13	14	15	16	17	18
Deletion%	0.0	0.0	**30**	0.0	0.0	0.0	0.0	0.0	0.0	**13**	0.0	**42**	**42**	**42**	**42**	**42**	0.0	0.0
Mutation%	0.0	0.0	**3.5**	0.0	0.0	0.4	0.0	0.0	0.0	0.4	0.0	0.0	0.0	0.0	0.4	0.0	0.0	0.0
Methylation%	1.2	1.6	**5.6**	0.0	0.4	1.4	0.5	0.7	0.0	1.5	1.7	0.0	1.6	1.6	1.6	0.0	0.8	1.6
Total%	1.2	1.6	**39**	0.0	0.4	1.8	0.5	0.7	0.0	**15**	1.7	**42**	**44**	**43**	**44**	**42**	0.8	1.6

Genetic (deletion and mutation) and epigenetic (methylation) changes in each CG site. Pronounced alternations of the CG hotspots are in bold. Genetic changes outside the CG sites were rare and negligible (data not shown). Deletion, but not mutation or methylation, was the most commonly identified alteration.

### Deletion Hotspots Are Not Due to Artifacts from Bisulfite-Treatment, PCR or Sequencing

As our focus on promoter assay will be based on the above sequencing results, we herein provide several pieces of data to ensure that the deletions were not artifacts of bisulfite-treatment of the DNA, PCR or sequencing. First, bisulfite modification reduced the GC content to ∼41% in the 222-bp *AMACR* promoter CGI, which made the sequencing easier to read; second, visual examination of sequencing chromatogram files showed clean and discrete peaks in the CGI region, indicating that the deletions we observed in bisulfite sequencing were not due to a GC compression artifact (Figure S2A). In addition, as an internal control to ensure complete bisulfite modification, we routinely examined and found that almost 100% of the non-CpG cytosines in this region were converted to T, indicating complete bisulfite modification.

To demonstrate that the CG12-16 deletion was not due to the PCR artifact, we used sequencing-verified plasmids with or without CG12-16 deletion as PCR templates, the PCR products showed expected size with different positions in 3% agarose gel (Figure S2B, left panel). Additionally, we used unmodified (not shown) and bisulfite-treated genomic DNA, with or without the CG12-16 deletion, as templates and performed multiple PCRs on the same two samples (Figure S2B, right panel). Results demonstrated the sizes of the amplicons derived from wild-type and deletion-variant templates were consistent, indicating that the deletion of CG12-16 was neither a PCR artifact nor a result of bisulfite-treatment.

Blast searches provide additional evidence that the CG12-16 deletion exists in the human genome, as of the two genome sequences, one is the reference assembly that corresponds to the sequence (NT_006576.15) without CG12-16 deletion and the other is the Celera assembly (NW_922562.1) exhibiting the deletion, which exactly matches what we discovered in the *AMACR* promoter in clinical samples (Figure S2C).

Finally, we conducted parallel bisulfite and regular sequencing on DNA isolated from LCM-captured normal or malignant colon epithelial cells from 9 colon specimens. Identical sequence results were obtained with the two methods (data not shown). Thus, in conclusion, these control experiments and *in silico* analyses demonstrate that the observed deletion hotspots in this CGI exist in colon tissues and are not results of artifacts generated from bisulfite-treatment, PCR or sequencing.

### Deletion of CG12-16 and Double CG3 and 10 Deletions Are Mutually Exclusive Molecular Events that Appear to Underlie AMACR Expression and CCa Development

We then investigated the relationship between deletion patterns in the *AMACR* promoter CGI and levels of AMACR expression (Table 2, left) in 55 iLCM samples to gain insight into how these deletions might affect gene expression *in vivo*. CG3-only deletions were rather common (13–41%) but were not correlated with AMACR expression, and CG10-only deletions were rare (0–5%). However, double CG3 and 10 deletions occurred at higher frequencies and invariably only in foci with no or little AMACR expression (17–28%, scores 0 and 1). In contrast, CG12-16 deletions were common (21–67%) and showed a positive correlation with the AMACR expression score. In total, foci with moderate and high AMACR expression (scores 2–4) had a high frequency of CG12-16 deletions (53–67%) and no double CG3 and 10 deletions.

**Table 2:** Deletion hotspots of *AMACR* CGI and their relation to the level of AMACR expression and colon histological entity.

CG deletion Pattern	Expression score					Histological entity					
	0	1	2	3	4	Normal	TA	VA	Well	Mod.	Poorly
**Δ3 only**	13	41	23	27	13	36	9.1	22	22	0.0	10
**Δ10 only**	0.0	5.0	0.0	0.0	0.0	0.0	2.3	0.0	0.0	0.0	3.6
**Δ3 & 10**	**17**	**28**	**0.0**	**0.0**	**0.0**	**24**	**25**	**0.0**	**0.0**	**0.0**	**0.0**
**Δ12-16 only**	**32**	**21**	**53**	**67**	**66**	**31** *	**39** *	**33** *	**56**	**89**	**14** *
**Δ3 & 12-16**	1.2	0.0	2.3	0.0	0.0	1.9	0.0	0.0	0.0	0.0	0.0
**Δ10 & 12-16**	0.0	0.0	0.0	0.0	0.0	0.0	0.0	0.0	0.0	0.0	0.0
**Δ3, 10 & 12-16**	0.0	0.0	0.0	0.0	0.0	0.0	0.0	0.0	0.0	0.0	0.0
**No changes**	37	5.9	22	6.8	22	6.4	25	46	22	11	72

Samples were divided into five groups according to their AMACR expression score (Left) or into six groups according to their histologic entity (Right). Left: Deletion at CG3 (Δ3 only) fluctuated as AMACR expression level went from 0 to 4; deletion at CG10 (Δ10 only) was a rare (≤5%) event in colon cells; Notably, CG3 and 10 double-deletions (Δ3 & 10, bold) were the only deletions identified in groups with low AMACR expression (score: 0–1). Frequent CG12-16 deletion (Δ12-16 only, bold) was correlated with high AMACR expression (53%, 67% and 66%, respectively. Score: 2–4). The rest of deletion combinations (Δ3 & 12-16; Δ10 & 12-16; and Δ3, 10 & 12-16) were not found or were at a low level (<2.5%). Right: Double-deletions at CG3 and 10 were found only in the normal and TA samples and notably absent in VA and CCas of all grades. CG12-16 deletion was found in all the sample groups but occurred at higher frequencies in well- and moderately differentiated cancers (56% and 89%, respectively). In contrast, in the poorly differentiated cancers, the sequence of the CGI was largely unchanged (72%) with only 14% deletion of CG12-16. Mutual exclusion of deletion at CG12-16 and double-deletion at CG3 and 10 (0%) is one of the features of the samples studied. Compared with the moderately differentiated group that has the highest deletion rate, significant difference of CG12-16 deletion was identified in the asterisk marked groups (^*^, *p*<0.05).

Next, we examined the type of deletions found in the six histological entities (Table 2, right) to determine their relationship to the adenoma-carcinoma progression paradigm. CG3-only deletions were commonly found among normal and CCa foci. In most cases, GC10 deletions occurred as double CG3 and 10 deletions found in normal epithelium and TA (24–25%). In contrast, the double-deletion was not identified in VA or in CCa of any grade. CG12-16 deletions were found in all six histological entities; however, their frequency markedly increased in well- (56%) and moderately (89%) differentiated cancers and correlated with high AMACR expression in these lesions (mean expression score ∼3; Figure 1E–G). It is of interest that the frequency of deletions of all kinds was low in poorly differentiated cancers; 72% of these foci have no lesions in the *AMACR* promoter CGI. Like all other CCas, they lack the deletion of CG3 and 10; the frequency of CG12-16 deletion in these CCas was low (14%), which correlates with negligible to low levels of AMACR expression in these lesions (mean expression score ∼0; Figure 1H). Compared with the CG12-16 deletion in the moderately differentiated group that has the highest deletion rate, statistic analysis indicated the deletion was significantly changed in the normal, TA, VA and poorly differentiated groups but not in the well differentiated group.

Together these data showed an intriguing *in vivo* phenomenon. Consistently, in all the samples analyzed, deletion of CG12-16 are not co-existed with double CG3 and 10 deletions (frequency = 0; Table 2). Additionally, double-deletions at CG3 and 10 are found only in normal epithelium and TA and are not observed in VA and CCa of all grades. In contrast, CG12-16 deletions are associated with moderate and well differentiated CCa that express high AMACR but not in poorly differentiated cancers that show negligible AMACR expression. These findings provide the impetus for a study of the effects of these deletions on *AMACR* transcription an *in vitro* system (the HCT 116; see below).

### Deletion of CG12-16 Is a Polymorphism But the Deletions at CG3 and CG10 Are Somatic Lesions

To better understand the relevance of these deletions to colon carcinogenesis, we must ask if these deletions are results of genetic events occurring in somatic cells of the colon or are constitutional alleles exist in the general population. Before this study, the only information available is that a sequence (NW_922562.1) harboring the CG12-16 deletion in the Celera assembly (Figure S2C). No *AMACR* sequences with deletion at CG3 or CG10, or at both sites have been reported in genomic databases.

We used randomly sampled genomic DNA isolated from whole blood of 96 individuals (48 males and 48 females) from a relatively homogeneous Caucasian population of northern German for our study [28]. A 173 bp region encompassing all 18 CG sites within the *AMACR* promoter CGI were analyzed by regular and bisulfite sequencing (Figure 2B). The CG12-16 deletion was found to be a sequence variant with an allele frequency of 43% in the population (Table 3 and 4). The observed genotype frequencies conform to the expectations of Hardy-Weinberg proportions (Table 3, *p*>0.05). Between male and female samples, chi-square test for the genotype difference and allele frequency differences are not statistically significant (*p*>0.05). In contrast, in these blood DNA samples, no other deletions or mutations were found at any of the other CG sites in this region of the *AMACR* CGI, including CG3 and CG10.

**Table 3:** The distribution of CG12-16 deletion polymorphism.

Gender	Homozygous for wild type alleles (# case)	Heterozygous for deletion of CG12-16 (# case)	Homozygous for deletion of CG12-16 (# case)	Allelic frequency (%)
Male	19	20	9	40
Female	13	25	10	47
Total	32	45	19	43

The 96 blood genomic DNA samples were from general individuals in a relatively homogeneous Caucasian population of northern German. Hardy-Weinberg equilibrium test showed within the males and females of this population, the distribution of genotype frequencies follows H-W expectation (*p* = 0.37 and 0.75, respectively); Chi-square test showed between male and female samples, the genotypic difference (*p* = 0.42) and the allelic difference (*p* = 0.31) are not statistically significant. In this population, the frequency of the CG12-16 deletion allele is 43%.

**Table 4:** The distribution of deletion, mutation and methylation in *AMACR* promoter CGI in whole blood DNA samples.

CG site	1	2	3	4	5	6	7	8	9	10	11	12	13	14	15	16	17	18
**Deletion%**	0.0	0.0	0.0	0.0	0.0	0.0	0.0	0.0	0.0	0.0	0.0	**42.9**	**42.9**	**42.9**	**42.9**	**42.9**	0.0	0.0
**Mutation%**	0.0	0.0	0.0	0.0	0.0	0.0	0.0	0.0	0.0	0.0	0.0	0.0	0.0	0.0	0.0	0.0	0.0	0.0
**Methylation%**	2.8	0.0	**16.7**	2.8	0.0	0.0	0.0	0.0	0.0	**11.1**	0.0	0.0	0.0	0.0	0.0	0.0	5.6	2.8
**Total%**	2.8	0.0	**16.7**	2.8	0.0	0.0	0.0	0.0	0.0	**11.1**	0.0	**42.9**	**42.9**	**42.9**	**42.9**	**42.9**	5.6	2.8

No mutation was identified in the samples. Deletion hotspot (bold) was identified only at CG12-16, whereas the methylation hotspots (bold) were identified at CG3 and CG10.

Interestingly, although deletions/mutations at CG3 and/or CG10 were not found by normal sequencing, bisulfite sequencing demonstrated that the two CG sites are methylation hotspots in blood DNA samples, exhibiting a prevalence of 16.7% and 11.1%, respectively (Table 4). These frequencies were higher than those observed in tissue samples in which deletion is the predominant type of lesion at these two sites (Table 1). The fact that both single and double deletions at CG3 and CG10 are completely absent in blood samples but occur at frequencies between 13–30% in colon tissue DNA indicates that they are somatic lesions.

### Deletion of CG12-16 and Double-Deletion of CG3 and 10 Exert Opposite Actions on *AMACR* Transcription

**Figure 4:**
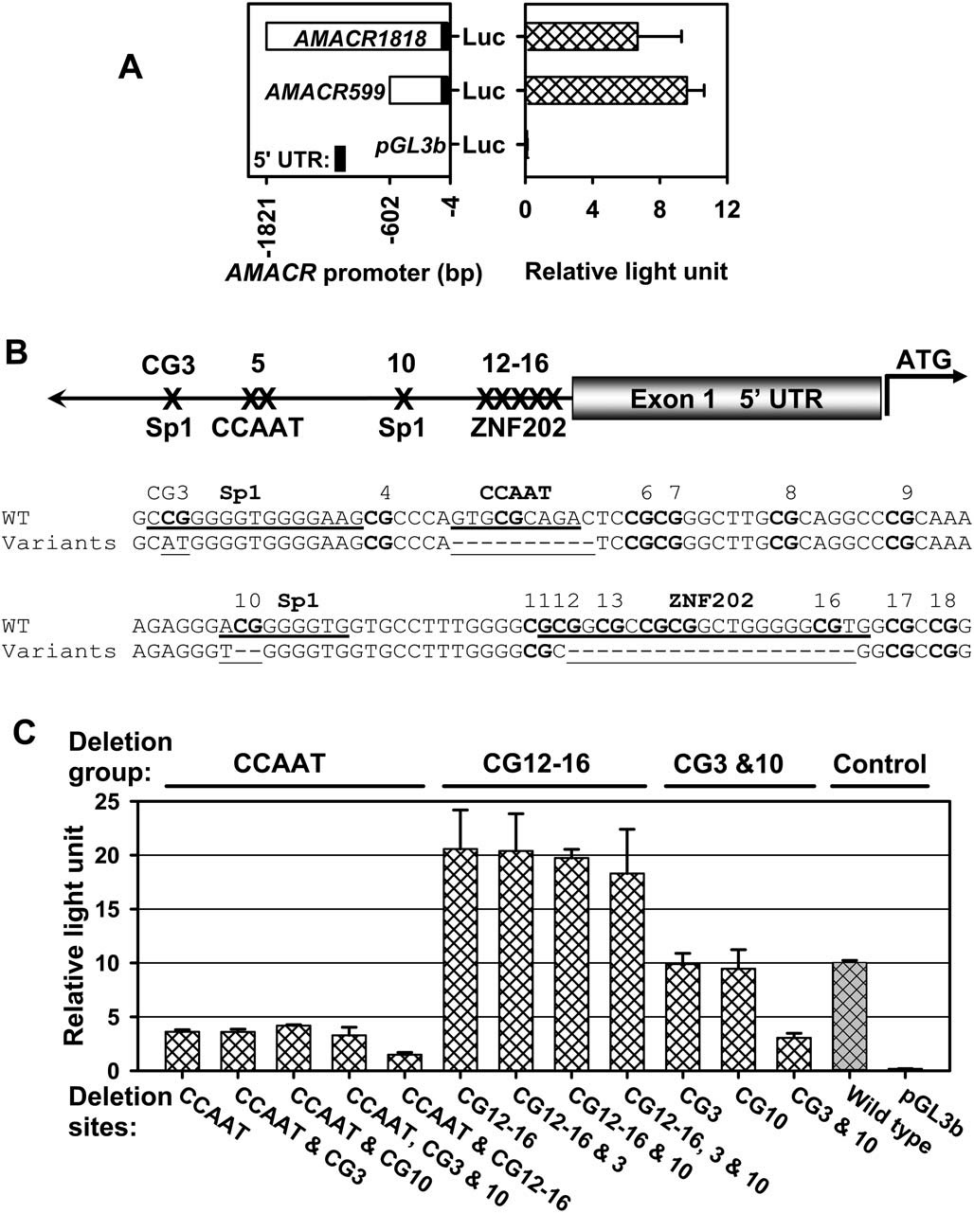
Deletion hotspots in *AMACR* promoter CGI are the *cis*-acting elements. A: Promoter assays showed that *AMACR599* with the CGI in it had promoter activity comparable to that of *AMACR1818*, suggesting that the 599 bp region is critical for the gene regulation. Thus, we selected *AMACR599* for further investigation. The promoter activity was normalized as relative light units. B: The location of the deletion hotspots in *AMACR* promoter. Various sequence variants were compared with the wild-type promoter. A previously identified CCAAT box is illustrated. C: Compared with the wild-type *AMACR599*, deletion of CCAAT enhancer element at CG5, or in combination with other deletion hotspots at CG3, 10 and 12-16, significantly reduced the promoter activity (58∼67%, *p*<0.0001, one-way analysis of variance, followed by Tukey's HSD *post hoc* test). No significant differences among the CCAAT deletion groups (*p* = 0.014 to 1) were observed. When the CCAAT enhancer was maintained intact, the single deletion at CG12-16 or in combination with deletion hotspots at CG3 and 10 resulted in an increase in the promoter activity by 83−105% (*p*<0.0001) but no significant difference among the deletion groups (*p* = 0.060 to 1). Further, when the CCAAT enhancer was maintained and CG12-16 was intact, deletion of either CG3 or CG10 did not change the promoter activity significantly (*p* = 0.26 and 0.69, respectively). In contrast, double-deletion at CG3 and 10 decreased the promoter activity by 69% (*p*<0.001).

To determine whether the *in vivo* deletions affect *AMACR* gene transcription, we first established that the human CCa cell line HCT 116 is a suitable model for *AMACR* promoter study *in vitro*. These cells express *AMACR* transcripts, have an intact promoter sequence with an unmethylated CGI (data not shown), and therefore should have an intact “transcriptional machinery,” including transcription factors for *AMACR* expression. Real-time RT-PCR showed that this cell line expresses both *Sp1* and *ZNF202* at significant levels. We cloned a long (1,818 bp; −1821/−4) and a short (599 bp; −602/−4) 5′ *AMACR* promoter sequence, both containing the newly identified CGI, into pGL3b reporter vector (Figure 4A). The two sequences showed comparable promoter activities in HCT 116 cells. These data suggest the localization of core promoter elements within the 599-bp sequence (*AMACR599*), which was used to derive all other mutants in this study.

To directly demonstrate that the deletion hotspots affect gene transcription, we generated deletion and/or mutation mutants of *AMACR599* by targeting single or multiple sites (Figure 4B). Since a previous study reported gene-regulatory activity of the CCAAT enhancer aligned to CG5 [5], we also included deletion mutants targeting this sequence in our study. Reporter assays performed in HCT 116 cells showed that deletion of the CCAAT enhancer sequence at CG5 led to a marked reduction in promoter activity (∼60%) regardless the integrity of CG3, CG10, or CG12-16 (Figure 4C). However, in the presence of an intact CCAAT enhancer, deletion of CG12-16, in the absence or presence of CG3, CG10, or double CG3 and 10 deletions, resulted in augmentation of promoter activity (∼100%). In contrast, deletion of CG3 and 10, but not a single deletion of either CG3 or CG10, caused a significant loss of promoter activity (∼60%). These findings indicate that deletion of CG12-16 and double-deletion of CG3 and 10 exert opposite actions on *AMACR* transcription.

To demonstrate that these regulatory mechanisms are not limited to CCas, we transfected these mutants into two PCa cell lines (PC-3 and LNCaP) and similar data were obtained (data not shown).

### Deletion Hotspots Are Located in *cis*-Elements Previously Not Known to Regulate *AMACR* Gene Expression

We next sought to understand how these deletions affect *AMACR* gene transcription. *In silico* analyses suggest the localization of Sp1 binding sites at CG3 and CG10 and a non-canonical ZNF202 binding site within the CG12-16 region (Figure 2B). However, it should be noted that *in silico*-based prediction requires experimental confirmation since recent ChIP-chip results have demonstrated a weak match between many consensus sequences and *in vivo* binding sites for specific transcription factors (TFs) [29],[30]. Poor correlations could be due a high degree of degeneracy for some motifs and/or the participation of other proteins at the binding sites. A series of confirmation studies were therefore performed to support our *in silico*-based predictions. We predict that deletion at CG3 or CG10 affects one of the two putative Sp1 binding sites, and deletion at CG12-16 impede occupancy of a ZNF202 protein to its *cis-*element located between CG12-16 (Figure 2B).

**Figure 5:**
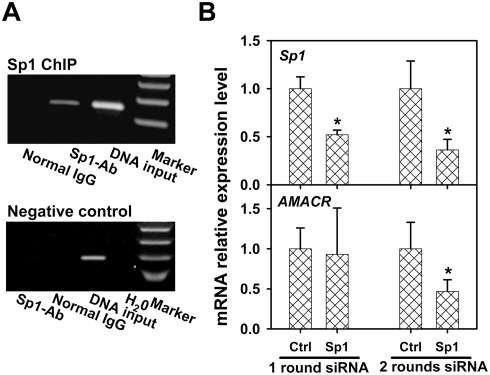
Transcription factors Sp1 is involved in *AMACR* gene regulation in HCT 116 cells. Putative Sp1 binding site at CG3 and 10 were identified. A: ChIP assay with Sp1 antibody targeting *AMACR* CGI (Figure 2B). A PCR signal was detected in the Sp1 antibody ChIP with genomic DNA and normal IgG-immunoprecipitated DNA as the PCR input and negative control, respectively (Figure 5A, top panel). As a ChIP negative control, amplification of a region in the last exon of *AMACR* gene distant to the putative Sp1 sites was included in the experiment. Only the DNA input showed the amplification (Figure 5A, lower panel). B: siRNA-mediated *Sp1* knockdown decreased the *AMACR* transcript level. Real-time RT-PCR demonstrated that the first-round siSp1 decreased the *Sp1* transcript level 48% (*p*<0.001). With the second-round siSp1, the *Sp1* transcript level further decreased 64% (*p*<0.001). In parallel, the first-round siSp1 resulted in little change in *AMACR* mRNA level (*p* = 0.66). Notably, the second-round siSp1 decreased the *AMACR* transcript level 53% (*p* = 0.002). In the negative control experiments, the same set of cDNA was used and siRNA knockdown of *Sp1* did not affect *GUSB* and *PP1A* gene expression (data not shown).

Using nuclear extracts from HCT116 cells, chromatin immunoprecipitation (ChIP) experiments were performed. Sp1 was found binding to a 174-bp sequence (−234/−60) that contains the two putative Sp1-sites at CG3 and CG 10 (Figure 5A, upper panel) but not to a 169-bp sequence (19553/19721) located in the last exon of *AMACR* (Figure 5B, lower panel). Small interfering (si)RNA-mediated *Sp1* knockdown decreased *AMACR* mRNA expression at the second-round of transfection (Figure 5B) but did not reduce transcript levels of *glucuronidase β* (*GUSB*) or *cyclophilin A* (*PPIA*), two unrelated genes (data not shown), in HCT 116 cells.

**Figure 6:**
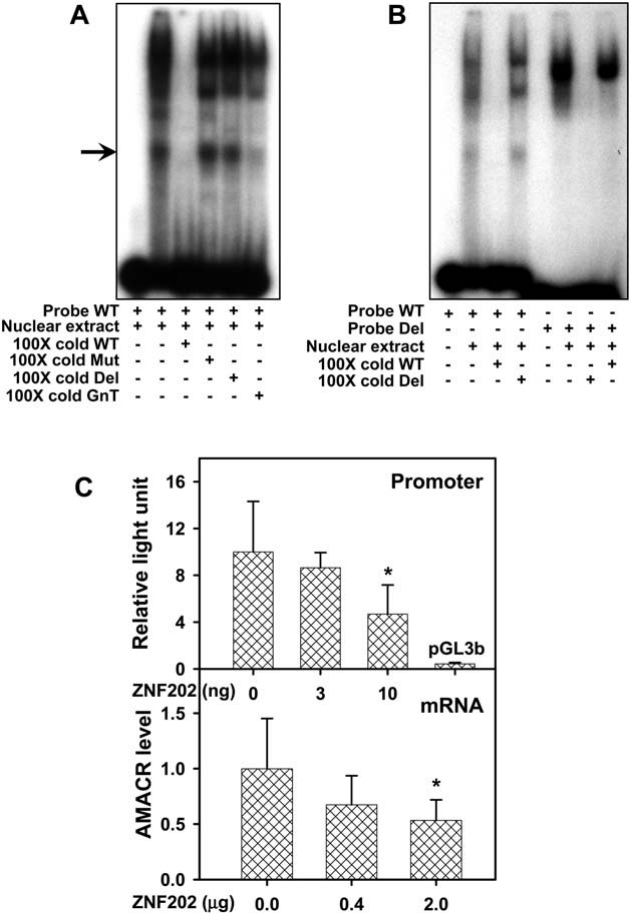
ZNF202 is involved in *AMACR* gene regulation in HCT 116 cells. A: The ^32^P labeled wild type (WT) probe corresponds to a sequence containing CG12-16 and its flanking regions (Table 5). Gel shift assays showed a single, specific shifted band (arrow) whose signal intensity could be impeded by co-incubation with 100× excess cold WT probe or a ZNF202 consensus sequence (GnT) but not by 100× excess mutated (Mut) or CG12-16 deleted (Del) ODNs. The other shifted bands represent unknown protein-DNA complexes formation. B, Left: Using labeled WT as a probe three major shifted bands were identified. Signal intensities of these bands could not be reduced by co-incubation with excess cold Del ODN that has deletion of CG12-16. Right: Using labeled Del as probe, one major band that differs from those observed with the labeled WT was identified. Its signal intensity was not diminished by co-incubation with excess cold WT. C: Ectopic expression of ZNF202 decreased *AMACR* promoter activity and mRNA level in a dose-dependent manner. Co-transfection of the ZNF202 expression plasmid with *AMACR599* (10 ng) decreased the promoter activity (*p* = 0.007); in parallel, ectopic expression of ZNF202 (2 µg) decreased the level of *AMACR* mRNA (*p* = 0.009). The asterisks indicate a significant difference in the group compared with the control.

Since there is no commercially available ZNF202 antibody for ChIP, gel shift assays were performed to assess HCT 116 nuclear protein binding to the putative ZNF202 binding site located within CG12-16 of the *AMACR* CGI. As can be seen in Figure 6A, one specific protein–DNA complex (arrow) was formed on the 45-bp ^32^P-labeled double-stranded oligonucleotide (ODN) encompassing CG12-16 and its flanking sequences (Probe WT). The formation of this complex could be impeded by 100-fold excess of unlabeled WT or a 26-bp ZNF202 consensus sequence (GnT; [27]). However, it is resistant to competition by 100-fold excess of a 45-bp mutant with the ZNF202 core sequence [31] mutated (Mut) or a 32-bp ODN devoid of CG12-16 (Del). Interestingly, protein-DNA complex formation patterns on labeled WT and Del were different with notable absence of the lower band that could be competed off by excess cold WT or GnT (Figure 6B). Finally, ectopic expression of ZNF202 induced a dose-dependent reduction of *AMACR599* promoter activity and concordant lower levels of *AMACR* mRNA (Figure 6C).

**Table 5:** Primers and oligonucleotides used in this study.

Assay	Primer	Sequence: 5′→3′
Bisulfite sequencing (*AMACR* CGI)	AM-bisF1	GGTAATAGGTAGGAGTTTTAAAGGTTAGTT
	AM-bisR1	AAAACTAAACAACCCTTAACCCCAACC
	AM-bisF2	TTGTAATTTAAGATTTAGGAATTTAGGTTG
	AM-bisR2	ACAACCTACAAAAAACCCTCCCAATC
Regular sequencing (*AMACR* CGI)	AM-F1	CTGGGGATCGCCCTGGTACA
	AM-R1	ACAGCTCCACGACCGAGATG
	AM-F2	AGAGGACGGTAACAGGCAGGAG
	AM-R2	AGGAAACTGAGCAGCCCTTAGC
Polymorphism study	PolyF	CAACCTACTGCATTTGGCACTG
	PolyR	CTGCAAGAAGCCCTCCCAAT
Promoter construction (*AMACR*)	pAM-F1	ACTCGAGGTTTTGATTTGCATTTCCCTGA
	pAM-R0	GAAAGCTTCCCAGTGCCCCGCTGAA
	pAM-F2	ACTCGAGTTCCTAGTGTAGTCTAAACT
ZNF202 (expression)	NotIZ202	TTGCGGCCGCTACAGCCGTGGAACCAGAGGA
	Z202ApaI	TTGGGCCCTAGGAGGTCTTTTCTGAGTGGGTCCT
ChIP (Sp1)	Sp1-IPf	AGCAACCTACTGCATTTGGCACTG
	Sp1-IPr	CTGCAAGAAGCCCTCCCAATC
ChIP negative control	ChIPnegF	GGCCTTTTGTCTTGGTGTTCAT
	ChIPnegR	CGTAGTGAGCCAACACATTTCC
Probes for gel shift assay	WT	TTGGGGCGCGGCGCCGCGGCTGGGGGCGTGGCGCCGGGGATTGGG
	Mut	TTGGGGCGCGATATTACGATTAAAAACGTGGCGCCGGGGATTGGG
	Del	TTGGGGCGCGGCGCCGGGGATTGGGAGGGCTT
	GnT	GTTGGTGGGGTGGGGGTGGGGGTGCC
Real-time RT-PCR (*Sp1* and *AMACR*)	Sp1f	CCAGGCCTCCAGACCATTAACC
	Sp1r	GGCATCTGGGCTGTTTTCTCCT
	AMf	GGGCCCGTTCTGTGCTATGGT
	AMr	TGGGCCCAGCTGGAGTTTCTC
Real-time RT-PCR negative control	GUSBf	AAACGATTGCAGGGTTTCAC
	GUSBr	CTCTCGTCGGTGACTGTTCA
	PP1Af	TTCATCTGCACTGCCAAGAC
	PP1Ar	TCGAGTTGTCCACAGTCAGC

*: Underlined: Promoter construction and ZNF202 expression, restriction sites for cloning; Gel shift assay probe WT, putative ZNF202 core sequence (MatInspector) flanking the CG12-16 region; Mut, mutated WT probe in the putative ZNF202 core sequence with C to T and G to A substitution; GnT, ZNF202 GnT consensus sequence in the *apoAIV* promoter region [27].

In sum, these findings provide evidence in support of CG3 and CG10 as Sp1 binding sites and CG12-16 as a ZNF202 *cis*-element. Sp1 and ZNF202 appear to regulate *AMACR* expression in an opposite manner.

## Discussion

The main objective of this study was to elucidate the regulatory mechanism underpinning *AMACR* gene expression in relation to CCa development. We identified a novel CGI upstream the translation start site in the proximal core promoter of *AMACR*. Although aberrant methylation of promoter CGIs is a common cause of transcriptional deregulation of genes involved in tumorigenesis [32], we found that *AMACR* activation did not occur by this mechanism during colon carcinogenesis. Instead, we found that two non-random, mutually exclusive *in vivo* events, involving a double-deletion at CG3 and 10 and the deletion of CG12-16, play essential but opposite roles in the process. Additionally, we discovered the differential “origins” of these two *in vivo* deletions by comparing sequencing data from blood DNA in a general population and those from LCM-microdissected colon samples. The deletion of CG12-16 in the *AMACR* 5′ CGI was found to be a constitutional allele with a frequency of 43% in a general population. In contrast, deletions at CG3 and/or CG10 were not observed in the blood samples indicating that these are genetic events occurring in somatic cells of the colon.

We observed a strong positive correlation between AMACR expression and the sequence of adenoma-carcinoma progression, suggesting a promotional function of AMACR in colon carcinogenesis. This postulate agrees with recent studies reporting that siRNA-mediated knockdown of *AMACR* mRNA or inhibition of the enzyme activity effectively curbed the growth of PCa cells [3],[4]. Intriguingly, both gene expression and CCa progression were closely correlated with the status of two mutually exclusive deletions found in the iLCM samples. Specifically, the double CG3 and 10 deletion was found only in histologically normal colonic glands and TAs that had negligible to absent AMACR expression and was absent in VA or CCas of all grades that had variable levels of AMACR expression. More important, the simultaneous deletion of these two sites effectively negated *AMACR* transactivation in HCT 116. We therefore propose that deletion at CG3 and 10 may effectively obviate colon carcinogenesis, possibly by impeding AMACR expression *in vivo*. In this regard, adenomas harboring double-deletions of CG3 and 10 might have a low likelihood of development to CCas; its potential diagnostic value merit further study.

In contrast to CG3 and 10 double-deletions, deletions of CG12-16 were highly prevalent in well- and moderately differentiated CCas that strongly expressed AMACR. This finding is impressive because it stands in stark contrast with the classical view that deletions cause functional inactivation of genes. In this instance, the CG12-16 deletion in the *AMACR* promoter CGI behaves like a “gain-of-function” deletion. When viewed in this context, the CG12-16 deletion may be part of the sequential genetic changes that occur in tumor suppressors, DNA repair genes, and oncogenes during the development of CCas from adenomatous lesions [33].

Unlike the better differentiated CCas, most poorly differentiated cancers had a low percentage of deletions at CG12-16 and lacked AMACR expression. These cancers also had very few other aberrations including the double-deletion at CG3 and 10, in their *AMACR* promoter CGI. Because the gene is not silenced by DNA methylation, or by irreversible genetic events such as deletions, we have to consider the possibility that these cancers may have a clonal origin different from that of the better differentiated carcinomas. Alternatively, during their evolution, these cancers may acquire a metabolic phenotype that is independent of AMACR overexpression.

Much could be learned about the relationship between the CG12-16 deletion polymorphism and CCa risk by comparing the allelic frequency of this sequence variant in blood samples (Table 3 & 4) to those found in LCM-captured histological entities of the colon (Table 1). The overall allelic frequency of this deletion amongst the 239 alleles from the various histological entities of the colon was found to be ∼42% (Table 1, row 1), which matches the allele frequency observed in the blood samples is ∼43%. This suggests that there may not be additional somatic events altering the frequency of this constitutional sequence in the colon. Yet, the prevalence of this lesion reaches 89% in the moderately differentiated CCas, which showed significant difference (*p*<0.05) compared to the normal, TA, VA and poorly differentiated but not the well differentiated samples. Collectively, these data adds up to the hypothesis that individuals with the CG12-16 deletion variant are more likely to develop CCas that are well or moderately differentiated. Conversely, those carrying the wild type variant may be more prone to develop poorly differentiated CCas. Clearly, such a provocative hypothesis would have to await a well-designed population study for confirmation.

Contributing significantly to our understanding of how *AMACR* is regulated, we here provide the first evidence that deletion hotspots in the *AMACR* promoter CGI correspond to *cis*-elements for Sp1 and that this transcription factor regulates AMACR expression. Our data support the regulation of *AMACR* by Sp1, as ChIP assays showed Sp1 binding to a region of the *AMACR* promoter CGI containing the predicted sites and siRNA-mediated *Sp1* knockdown decreased *AMACR* mRNA levels in HCT 116. Reporter assays revealed that single deletion at either CG3 or CG10 did not affect *AMACR* transcription, whereas the double-deletion significantly abrogated the promoter activity, suggesting that the integrity of one site at either CG3 or CG10 is sufficient to maintain the promoter activity.

In contrast, deletion of CG12-16 enhanced *AMACR* transcription, signifying the likely presence of a repressor binding site in this region. We did not perform a ChIP assay for ZNF202 because no antibody was commercially available for the immunoprecipitation. However, results from gel shift assays were highly suggestive of the existence of a non-canonical ZNF202 binding site within this sequence. Our finding that ectopic overexpression of ZNF202 reduced *AMACR* promoter activity lends credence to this notion. However, we are aware of the fact that these data did not provide the definitive evidence that the CG12-16 sequence contains a ZNF202 *cis*-element, which still awaits a formal demonstration in future investigations. It is always possible that some unknown transcription factors other than ZNF202 could be involved in this regulation.

Intriguingly, ZNF202 is a transcriptional repressor for genes affecting the vascular endothelium as well as lipid metabolism. We have examined the promoters of five other ZNF202 target genes [27],[34],[35] (*apoA4*, *apoE*, *lecithin cholesterol acyltransferase*, *lipoprotein lipase*, and *phospholipid transfer protein*) and did not find deletions or other aberrations in their ZNF202 *cis*-element in colon and prostate cancer cells (unpublished data). Thus, the activation of *AMACR* via deletion of a ZNF202 *cis*-element would be a phenomenon unique to *AMACR* gene regulation, if the CG12-16 sequence was shown to house this element. Several epidemiologic and animal studies have observed associations between the risk of metabolic syndromes/coronary heart diseases and the prevalence of colon adenomas/carcinomas [36]–[38]. Perhaps the loss of ZNF202-mediated repression of specific target genes, including *AMACR*, is a common cause of these diseases. Apropos of this view, carcinogenesis is being recognized increasingly as a metabolic disorder characterized by a shift from glycolysis to fatty acid utilization as the energy source fueling cell growth [2].

Finally, deletion of the CCAAT enhancer resulted in the loss of promoter activity regardless of the status of other elements, indicating that CCAAT enhancers are part of the basal transcriptional complex for *AMACR*. However, we did not find alterations in this *cis*-element in the iLCM samples, suggesting that such alterations do not contribute to aberrant expression of AMACR during colon carcinogenesis.

At present, it is unclear how the deletions at in the *AMACR* promoter arise. However, first, we noticed that deletion hotspots at CG3 and CG10 are also methylation hotspots (Table 1 and Table 4). It has been reported that methylated CG sites are mutation hotspots [39] as suggested in Figure S3A. Second, scrutiny of the CGI sequence revealed two 7 nt direct repeats (Figure 2B). We postulated that forward slipped-strand mispairing [40],[41] of the repeats, may result in the CG12-16 deletion during DNA replication (Figure S3B). If this mispairing happens, such slippage will cause the exact 20 bp deletion found in *AMACR* promoter. These proposed mechanisms speculated to be responsible for these deletions will of course have to await future experiments for corroboration.

Collectively, we identified two major types of *in vivo* deletions in the *AMACR* promoter that appear to modulate gene expression and may play contrasting roles in carcinogenesis. In essence, a double-deletion at CG3 and 10 prevents AMACR overexpression and may impede colon carcinogenesis. In contrast, carriers of sequence variants with or without the CG12-16 deletion may have different propensity to develop well/moderately differentiated CCas versus the poorly differentiated cancers. Finally, our data suggest that these deletion hotspots are *cis*-elements for Sp1 at CG3 or CG10 and for ZNF202 at CG12-16. The proposed mechanisms for *AMACR* promoter regulation and the deletion hotspots provided important platforms for the further study of *AMACR* gene deregulation during carcinogenesis.

## Materials and Methods

### Samples

Archival specimens were obtained from the Department of Pathology at the University of Massachusetts Medical School. Specimens from 35 cases were immunostained and microdissected to obtain the 55 iLCM samples: 11 TAs with mild dysplasia, 8 VAs, 6 well differentiated carcinomas, 6 moderately differentiated carcinomas, 7 poorly differentiated carcinomas, and 17 histologically normal colon tissues with 9 normal crypt and 8 apical surface epithelial samples. For the TAs, pronounced dysplastic changes, which often linked to positive AMACR, were uncommon. Most of the foci had mild dysplastic changes, and we focused our study of TAs on this type of sample. These samples were used for bisulfite sequencing analysis. Specimens for nine additional cases were obtained from the Pathology Department of the University of Cincinnati Medical Center and used to obtain nine LCM samples of normal epithelial, adenomatous, and carcinomatous cells for a regular DNA sequencing for comparison with bisulfite sequencing. Blood samples for polymorphism assay were from a relatively homogeneous Caucasian population of northern German [28]. The use of these samples was reviewed and approved by the respective institutional review boards at the two institutions.

### Immunohistochemistry and Laser-Captured Microdissection

Multiple sections were cut from each case specimen. One section was stained with hematoxylin and eosin (H&E) and used for identification of histologic entities. The others were immunostained for AMACR with the P504S antibody (Dako Cytomation, Carpinteria, CA) and lightly counterstained with hematoxylin as previously described [8],[42]. Areas representative of the histologic features and the overall intensity of AMACR expression found in a given case were identified in immunostained sections. These areas were then located in the replicate. The coverslips were then removed, H&E-stained, and microdissected as previously described [43].

### Evaluation of AMACR Protein Expression

Each of microdissected foci was given a score (0–4) reflective of the level of AMACR expression. When uniformly intense immunostaining was observed in at least 95% of cells in the section, the level of AMACR expression was designated as very strong (score = 4). If staining was less intense, not uniform throughout the section, and in fewer than 95% of the cells, the level of expression was designated as strong (score = 3). If the intensity of stain was weak, not uniform, and in 50% or fewer the cells, the section was graded as medium (score = 2) or weak (score = 1). Cases were scored as negative (score = 0) when the section showed no staining.

### Bisulfite Sequencing and Regular DNA Sequencing Analysis

Genomic DNA was extracted from the LCM samples by DNeasy Blood & Tissue Kit (Qiagen, Valencia, CA) with 20 µg of yeast tRNA added as a carrier. DNA was bisulfite-modified with the CGenome DNA Modification Kit (Millipore, Billerica, MA). Sequencing service was provided by Macrogen (Seoul, Korea) with BigDye terminator used in a 96-capillary 3730xl DNA analyzer. Bisulfite-sequencing PCR-targeting *AMACR* promoter CGI was conducted by nested PCR. Primers AM-bisF1/AM-bisR1 and AM-bisF2/AM-bisR2 (Table 5 and Figure 2) were used in the first round and nested PCR, respectively. The targeting region was from −276 to −55, with the translation start site designated as +1. PCR was performed with platinum *Taq* (ABI/Invitrogen, Carlsbad, CA) for 38 cycles with the annealing temperature at 56°C and 57°C in the first and nested PCR, respectively. Amplified fragments were purified in 1% agarose gel, TA-cloned, and about five colonies were picked from each sample for sequencing. Regular sequencing of the same CGI flanking region was performed in parallel using unmodified DNA samples and the regular primers AM-F1/AM-R1 and AM-F2/AM-R2 (Table 5). Proper controls were included in all experiments to ensure that the findings were not confounded by incomplete bisulfite modification, PCR artifact, or sequencing errors.

### Polymorphism Study in *AMACR* Promoter

Blood genomic DNA for the polymorphism study was extracted by DNeasy Blood & Tissue Kit. Using 50 ng genomic DNA as template, the PCR was performed for 40 cycles in the presence of 5% DMSO by platinum *Taq* with PolyF/PolyR as the primers (Table 5 and Figure 2). The annealing temperature was set at 58°C. The expected PCR product encompassing CpG sites 1–18 without CG12-16 deletion is 173 bp in length. After gel purification, the PCR products were TA cloned and the plasmids in colonies were directly amplified for sequencing by the Rolling Circle Amplification Kit (GE Health Care, Piscataway, NJ). PCR products from alleles with the deletion of CG12-16 could also be visualized by a size difference from amplicons derived from wild type alleles in a 3% agarose gel. To determine the prevalence of methylation in this region of the *AMACR* promoter, aliquots of the extracted genomic DNA was subjected to bisulfite sequencing.

### Promoter Construction and 5′- and Site-Specific Deletions

The *AMACR* promoter region immediately upstream of the translation start site was amplified from genomic DNA of HCT116 cells. With forward primer pAM-F1 (Table 5, *Xho*I site underlined) and reverse primer pAM-R0 (*Hind*III site underlined) used in PCR, the resulting 1818-bp AMACR promoter (from −1821 to −4) was cloned into luciferase reporter vector pGL3b (Promega, Madison, WI) and designated as *AMACR*1818. The promoter sequence was verified by sequencing. A 5′ truncated promoter (designated as *AMACR*599, from −602 to −4) was generated by nested PCR with PAM-F2/PAM-R0 as the primers. Promoter site-specific deletion variants were obtained by using the Genetailor site-directed mutagenesis kit (Invitrogen). After sequencing, the promoter variants were released from the cloning vector and recloned into pGL3b.

### Cell Culture, Transfection, and Promoter Luciferase Assay

All reagents used for cell cultures, including heat-inactivated FBS, were obtained from Invitrogen. Human CCa cell lines HCT 116, SW480, SW620, and DLD-1 were obtained from the American Type Culture Collection (Manassas, VA). The cells were maintained in the same condition as HCT 116 cells, which are cultured according to the provider's recommendations. Unless specified, 6×10^4^ HCT 116 cells were plated one day before transfection in each well of the 24-well plate. The cells were transfected with a total of 0.2 µg of DNA, including 10 ng of cotransfected CMV promoter-driven *LacZ* gene (CMV-LacZ) as the internal control. Plasmids for transfection were purified with the EndoFree Plasmid Maxi Kit from Qiagen. Two microliters of Plus and 1 µl of Lipofectamine (Invitrogen) were used in the transfection according to the protocol. The promoter activity was analyzed as previously described [44].

### Chromatin Immunoprecipitation (ChIP)

The ChIP assay was performed with the EZ ChIP Kit from Millipore according to the manufacture's instruction. A total of 7.5 µg of anti-Sp1 rabbit polyclonal IgG (cat. no. 07-645, Upstate/Millipore) was used in each IP. Primers Sp1-IPf/Sp1-IPr targeting −234 to −60 CGI (Table 5) were used in PCR with platinum *Taq* in the presence of 5% DMSO with an initial denaturation at 94°C for 1 min, followed by 36 cycles of 94°C for 30 sec, 58°C for 30 sec, and 72°C for 15 sec. As a negative control for DNA IP, primers ChIPnegF/ChIPnegR targeting the gene's last exon were used in PCR (Table 5).

### Real-Time RT-PCR

RNA extraction, reverse transcription, and real-time PCR, together with the primers for *GAPDH* and 18S rRNA, were described previously [44]. The tested primers used to detect *AMACR* and *Sp1* transcripts were AMf/AMr and Sp1f/Sp1r, respectively (Table 5). As the siRNA control, primers for *GUSB* and *PP1A* gene were used in the real-time RT-PCR and listed in the Table 5. The relative level of gene expression was calculated by the 2^−ΔΔCt^ method as described in detail in our previous studies [44],[45].

### Small Interfering RNA-Mediated *Sp1* Knockdown

1.5×10^5^ HCT 116 cells were seeded at day -1 before transfection in each well of the 6-well plate. At day 0, transfection was performed with 5 µl of Lipofectamine 2000 (Invitrogen/ABI) and 7.5 µl of 20 µM siRNA per well according to the protocol. siSp1 (ON-TARGETplus SMARTpool, cat. no. L-026959-00, Dharmacon, Lafayette, CO) was used to knockdown *Sp1* expression with Non-Targeting siRNA (cat. No. D-001210-01-05) as the control. At day 3, the cells either were collected for real-time RT-PCR analysis or were split at 1.5×10^5^ cells per well. The second round of siRNA was performed on day 4 and analyzed on day 7. To demonstrate the specificity of siRNA knockdown effects, in parallel, the expression of two unrelated genes of *GUSB* and *PP1A* were analyzed.

### Ectopic Expression of Zinc Finger Protein 202 (ZNF202)

Full-length coding sequence of *ZNF202* m1 transcript [27] was amplified by primers NotIZ202 and Z202ApaI (Table 5) from LNCaP cDNA. The sequencing-verified fragment was subcloned into pcDNA4/His/Max A expression vector (Invitrogen). For real-time RT-PCR, the expression plasmid was transfected into HCT 116 in the 6-well plate with the Nucleofector Kit and Nucleofector II device from Amaxa (Gaithersburg, MD).

### Gel Mobility Shift Assay

Probe sequences were shown in Table 5. Complementary single-strand DNA oligos were annealed in 1×PCR buffer (20 mM Tris-HCl, 50 mM KCl, pH 8.4) in a water-filled heat block. The annealing mixture was heated at 95°C for 3 min and cooled to below 30°C in 1 hr to generate 50 µM double-strand oligo. The double-strand oligos showed a single and stronger band in 3% agarose gel, and located at a different position than the single-strand oligos (photos not shown). HCT 116 cells nuclear extract was prepared by Nuclear Extract Kit (Active Motif, Carlsbad, CA) according to the manufacturer's instructions. Three µg of nuclear extract (1 µl) was used in each binding assay at 18°C in 10 µl. The assays were carried out according to the protocol described in the Gel Shift Assay System (Promega) with the following modifications: Probe labeling was performed with 10 U T4 polynucleotide kinase (New England Biolabs, Ipswich, MA) and 2 µl [γ-^32^P]ATP (3,000 Ci/mmol at 10 mCi/ml, Perkin Elmer, Waltham, MA) in a total volume of 10 µl at 37°C for 20 min. Electrophoresis of DNA-protein complexes was resolved in 6% DNA Retardation gel (Invitrogen) using 4°C 0.5× TBE buffer at 250 V for ∼35 min. Dried gels were exposed to X-ray film at −80°C for ∼1 hr and the images were captured by a digital camera.

### Genbank Accession Numbers

Five newly identified sequences of *AMACR* promoter variants with deletion/mutation at CpG hotspots were deposited into the Genbank (http://www.ncbi.nlm.nih.gov/Genbank/). The accession numbers for these variants are from EF636492 to EF636496, which represent a CG3 deletion, a CG3 mutation, a CG10 deletion, CG3 and 10 double-deletions, and a CG12-16 deletion, respectively. In addition, the accession number for the *AMACR* promoter from the Genbank reference assembly and Celera assembly are NT_006576.15 and NW_922562.1, respectively. The transcript reference sequences are NM_014324.4 and NM_203382.1 for *AMACR*, NM_003455 for *ZNF202 m1*, NM_138473.2 for *Sp1*, NM_000181.2 for *GUSB*, and NM_021130.3 for *PP1A*.

### Bioinformatics Analyses

Extensive gene analyses were carried out with GeneCards (www.genecards.org). BLAST (www.ncbi.nlm.nih.gov/BLAST/) was used to compare the sequence against Genbank. CGI was identified by MethPrimer at http://www.urogene.org/methprimer/index.html. Gene exon and intron information was obtained from Blat (http://genome.ucsc.edu/). PCR primers, except for real-time RT-PCR negative control (Real Time Primers, Elkins Park, PA) and bisulfite PCR, were designed by Primer3 [46] at http://frodo.wi.mit.edu/cgi-bin/primer3/primer3_www.cgi. The sequencing data were analyzed by ClustalW at http://www.ebi.ac.uk/clustalw. Putative TF binding sites in *AMACR* promoter deletion hotspots were scanned by MatInspector [31]. MatInspector utilizes transcription factor knowledge base to locate putative TF binding sites in sequence and minimize the number of false positive hits, but requires further confirmation through wet-bench works. It defines the “core sequence” (Table 5 and Figure 2B) of a putative binding site as the consecutive highest conserved positions in the DNA binding site.

### Statistical Analyses and Hierarchical Cluster Analysis

The hierarchical cluster analysis was based on the average linkage principle, and the absolute number of co-occurrences of different CG deletions was based on the similarity measure. The differences in AMACR expression (Figure 1) in the microdissected foci were compared among the different histologic categories using a one-way analysis of variance (nonparametric), followed by Tukey's HSD *post hoc* test for comparisons of all classes of lesions against normal cryptal cells. The analysis of CG12-16 deletion among the different histologic categories (Table 2) was carried out by SAS Proc Genmod software that assuming a log link and robust standard error estimation. The program estimates and tests differences between groups with respect to the proportion of deletion. A generalized linear model of binomial proportions was analyzed to detect differences. In other experiments, a two-tailed, unpaired *t*-test was performed between two groups. Except else where mentioned, the columns with error bars in the figures represent mean±95% confidence interval. For the CG12-16 deletion polymorphism study, Hardy-Weinberg equilibrium was used to test if specific disturbing influences are introduced to the samples, and chi-square test was used to exam genotypic and allelic differences between male and female. In all the analyses in this paper, unless otherwise stated, *p*<0.05 was considered as statistically significant.

## Supporting Information

Figure S1 Cluster analyses of mutation and the overall aberrations in *AMACR* promoter CGI. The same approach was used as indicated in Figure 3. Mutation basically occurred at CG3 and 10 in the LCM-captured colon samples, whereas the overall aberrations of deletion, methylation and mutation were at CG3, 10, and 12-16.
(0.12 MB TIF)

Figure S2 Quality control of bisulfite treatment, PCR, and sequencing. A: Representative bisulfite sequencing result with *AMACR* promoter short deletion at CG12-16 as an example. No CG compression was observed in the sequencing chromatogram. The peaks are discrete with clean background. The Cs (highlighted in red) in the “wild-type” *AMACR* promoter sequence were converted to Ts, demonstrating the complete bisulfite modification and hypomethylation of the CGI. B, Left: Bisulfite-specific PCR with the wild-type and CG12-16 deleted *AMACR* promoter as the template. The templates were cloned from *AMACR* promoter with the sequence verified. W, wild-type DNA template; D: template with CG12-16 deletion; (-): No template control. M: DNA marker. B, Right: Multiple bisulfite PCR assays demonstrating consistent size differences from samples carrying alleles with or without a deletion of CG12-16. C: Both the wild-type *AMACR* promoter sequence and the CG12-16 deleted sequence were identified in human genome assembly. NT_006576.15: reference assembly; NW_922562.1: Celera assembly.
(1.28 MB TIF)

Figure S3 Putative deletion mechanisms at the CG hotspots. A: CpG methylation-mediated mutation involved in the deletion at CG3 and 10. CG3 and 10 are the methylation hotspots. Methylated C is the hotspot of modification or spontaneous deamination that may result in the deletion caused by repairing deficiency. B: Slipped-strand mispairing involved in the deletion at CG12-16. Two direct repeats of 7 nt (bold) were located downstream of the CG11 (underlined). Forward slippage, usually 2–3 bp within the direct repeats during DNA replication, leads to the 20-bp deletion.
(0.25 MB TIF)
