# Supplementary figures and images for: Deletion Hotspots in AMACR Promoter CpG Island Are cis-Regulatory Elements Controlling the Gene Expression in the Colon
Source: PLoS Genet. 2009 Jan 16;5(1):e1000334. doi: 10.1371/journal.pgen.1000334 (PMC2613032; doi:10.1371/journal.pgen.1000334)

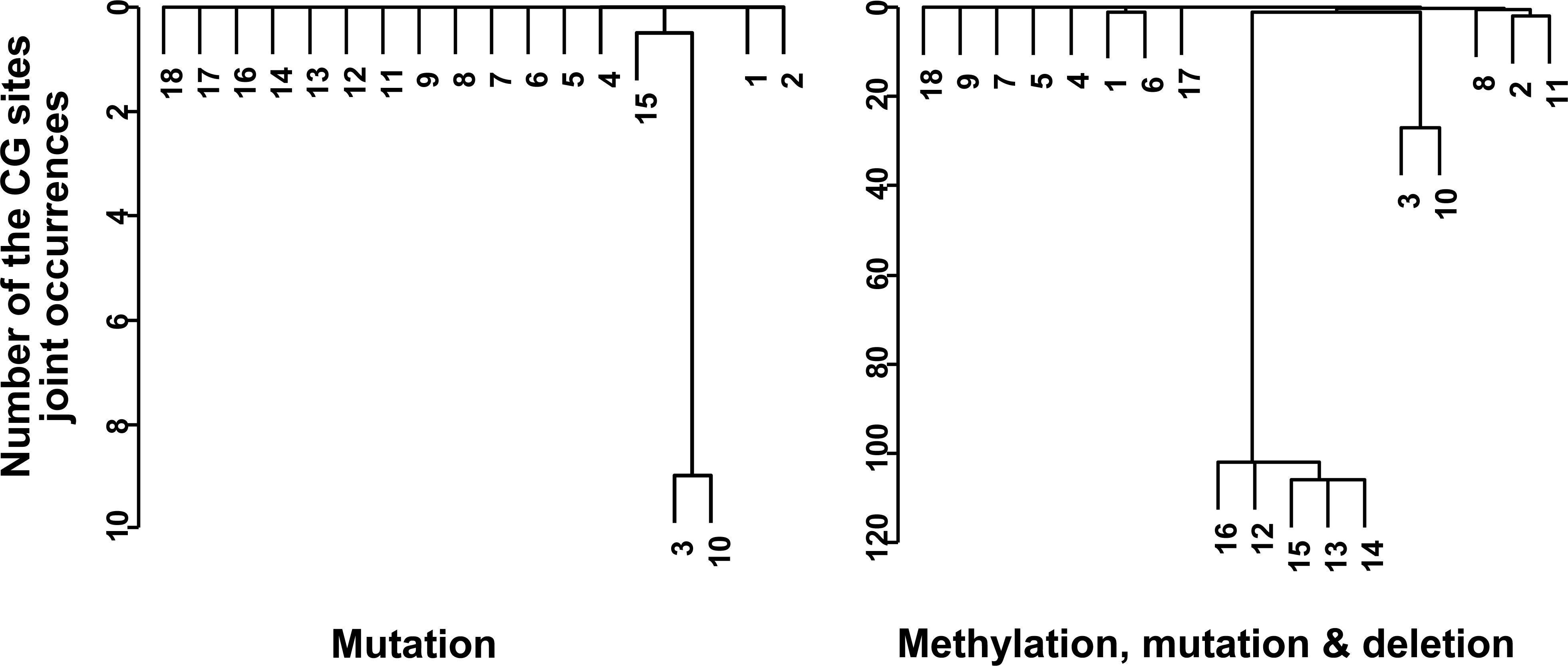

Supplement: Figure S1 — Cluster analyses of mutation and the overall aberrations in AMACR promoter CGI. The same approach was used as indicated in Figure 3. Mutation basically occurred at CG3 and 10 in the LCM-captured colon samples, whereas the overall aberrations of deletion, methylation and mutation were at CG3, 10, and 12-16. (0.12 MB TIF) [file pgen.1000334.s001.tif]

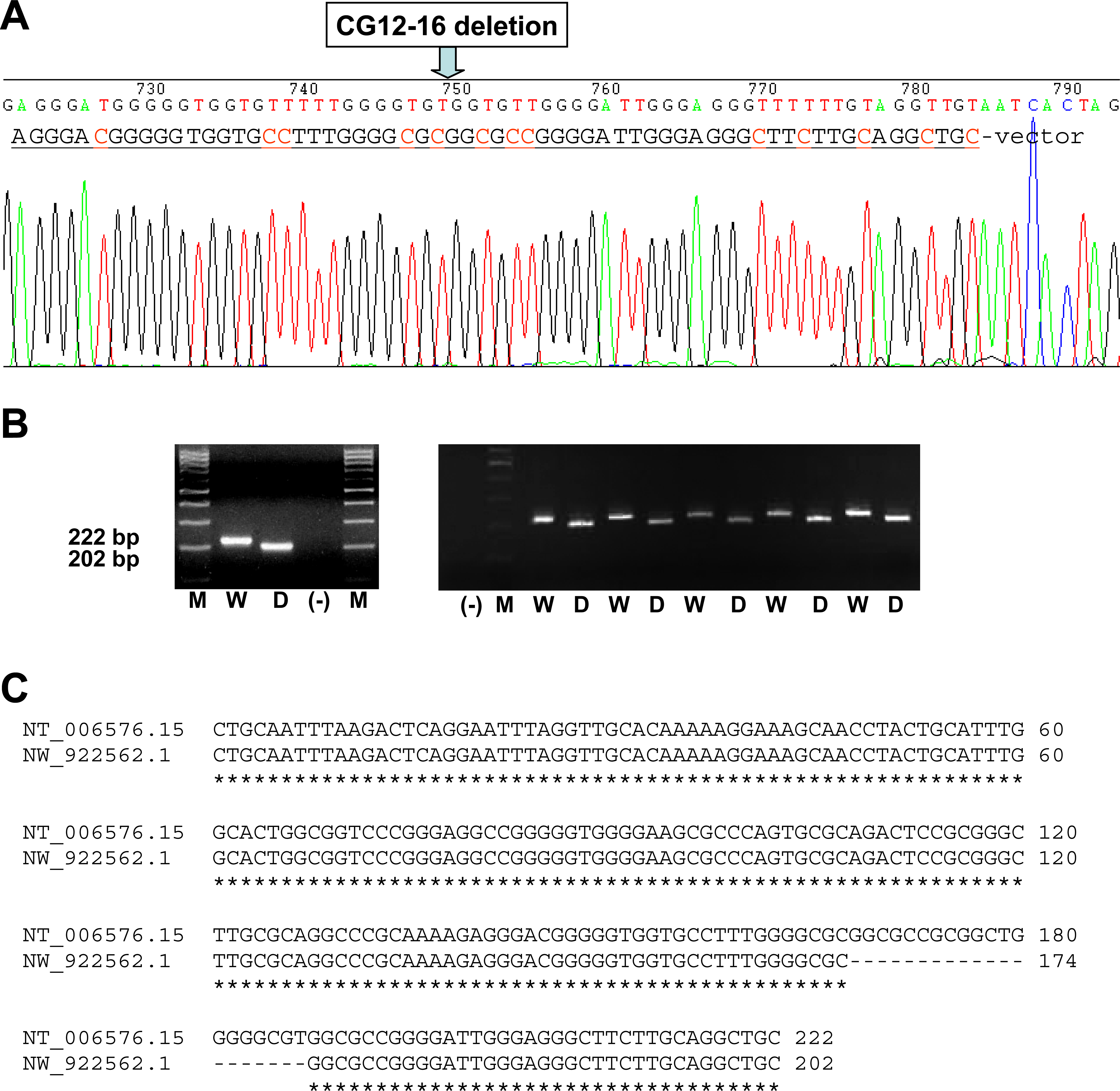

Supplement: Figure S2 — Quality control of bisulfite treatment, PCR, and sequencing. A: Representative bisulfite sequencing result with AMACR promoter short deletion at CG12-16 as an example. No CG compression was observed in the sequencing chromatogram. The peaks are discrete with clean background. The Cs (highlighted in red) in the “wild-type” AMACR promoter sequence were converted to Ts, demonstrating the complete bisulfite modification and hypomethylation of the CGI. B, Left: Bisulfite-specific PCR with the wild-type and CG12-16 deleted AMACR promoter as the template. The templates were cloned from AMACR promoter with the sequence verified. W, wild-type DNA template; D: template with CG12-16 deletion; (-): No template control. M: DNA marker. B, Right: Multiple bisulfite PCR assays demonstrating consistent size differences from samples carrying alleles with or without a deletion of CG12-16. C: Both the wild-type AMACR promoter sequence and the CG12-16 deleted sequence were identified in human genome assembly. NT_006576.15: reference assembly; NW_922562.1: Celera assembly. (1.28 MB TIF) [file pgen.1000334.s002.tif]

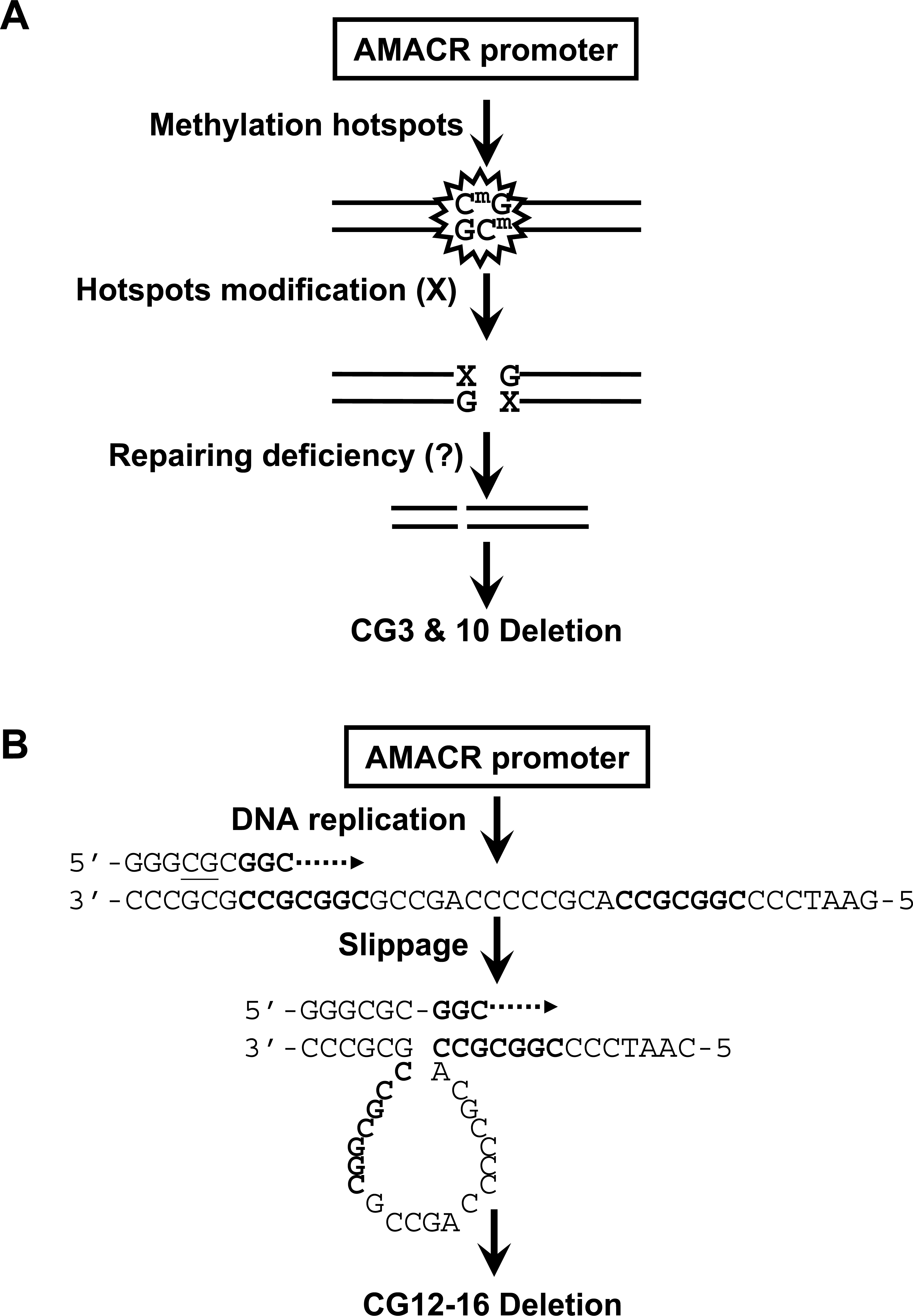

Supplement: Figure S3 — Putative deletion mechanisms at the CG hotspots. A: CpG methylation-mediated mutation involved in the deletion at CG3 and 10. CG3 and 10 are the methylation hotspots. Methylated C is the hotspot of modification or spontaneous deamination that may result in the deletion caused by repairing deficiency. B: Slipped-strand mispairing involved in the deletion at CG12-16. Two direct repeats of 7 nt (bold) were located downstream of the CG11 (underlined). Forward slippage, usually 2–3 bp within the direct repeats during DNA replication, leads to the 20-bp deletion. (0.25 MB TIF) [file pgen.1000334.s003.tif]
